# Pattern-based detection of anion pollutants in water with DNA polyfluorophores†
†Electronic supplementary information (ESI) available: Additional figures and tables and experimental details. See DOI: 10.1039/c4sc03992k


**DOI:** 10.1039/c4sc03992k

**Published:** 2015-02-18

**Authors:** Hyukin Kwon, Wei Jiang, Eric T. Kool

**Affiliations:** a Department of Chemistry , Stanford University , Stanford , California 94305-5080 , USA . Email: kool@stanford.edu ; Fax: +1 650 725 0259 ; Tel: +1 650 724 4741

## Abstract

Many existing irrigation, industrial and chemical storage sites are currently introducing hazardous anions into groundwater, making the monitoring of such sites a high priority. Detecting and quantifying anions in water samples typically requires complex instrumentation, adding cost and delaying analysis. Here we address these challenges by development of an optical molecular method to detect and discriminate a broad range of anionic contaminants with DNA-based fluorescent sensors. A library of 1296 tetrameric-length oligodeoxyfluorosides (ODFs) composed of metal ligand and fluorescence modulating monomers was constructed with a DNA synthesizer on PEG-polystyrene microbeads. These oligomers on beads were incubated with Y^III^ or Zn^II^ ions to provide affinity and responsiveness to anions. Seventeen anions were screened with the library under an epifluorescence microscope, ultimately yielding eight chemosensors that could discriminate 250 μM solutions of all 17 anions in buffered water using their patterns of response. This sensor set was able to identify two unknown anion samples from ten closely-responding anions and could also function quantitatively, determining unknown concentrations of anions such as cyanide (as low as 1 mM) and selenate (as low as 50 μM). Further studies with calibration curves established detection limits of selected anions including thiocyanate (detection limit ∼300 μM) and arsenate (∼800 μM). The results demonstrate DNA-like fluorescent chemosensors as versatile tools for optically analyzing environmentally hazardous anions in aqueous environments.

## Abstract 2

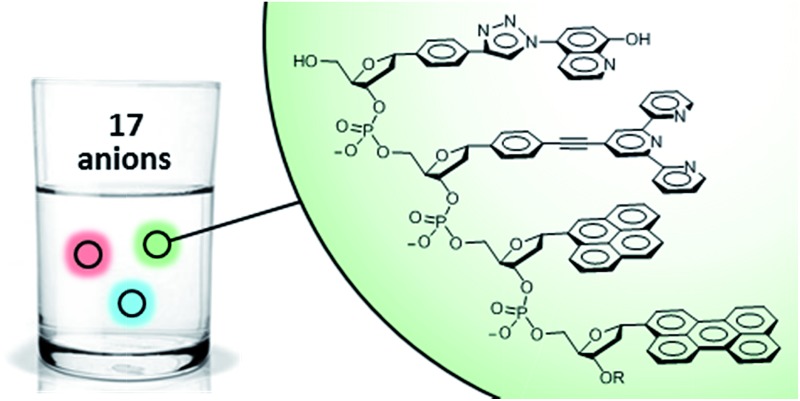
Eight fluorescent DNA-like oligomers bound to Y(iii) or Zn(ii) and attached to microbeads were able to distinguish 17 anions at micromolar concentrations in water.

## Introduction

Industrial, mining, refinery, and chemical storage sites pose risks of exposing harmful pollutants to the environment. Of the numerous toxic organic and inorganic species potentially generated at such sites, anion contaminants can be leached into groundwater and lead to environmental and health hazards. For example, chlorite, bromate and fluoride can be leaked from water treatment operations, perchlorate from military industries, and cyanide from mining.^1^ In addition, petroleum production also generates highly saline solutions containing multiple toxic anions.^2^ Another source of toxicity arises from irrigation in arid environments, which concentrates contaminants such as arsenate and selenate, resulting in human health hazards.^3^ Effective on-site monitoring of these anions requires methods compatible with low anion concentrations in aqueous media; however, typical instrumentation for anion analysis (such as ion chromatography)^4^ can be costly and usually requires transport of samples offsite to a central laboratory. To address these limitations, researchers are designing optical approaches to sensing that may be rapid and portable. Fluorescent anion chemosensors are under development recently, with goals of minimal sample preparation, high selectivity and sensitivity, novel emission mechanisms geared for specific sensing tasks, and possible miniaturization of instrument optics.^5^ Other optical detection methods are also under investigation, including the use of chromogenic sensors.^6^

Despite the growing field of optical anion sensing, detection and discrimination of a large number of hazardous anions from one another remains a challenge. Typical molecular probes are designed to recognize only one specific analyte^2,7^ and may not have been tested for specificity against a wider array of related anions. Another common limitation is a requirement for organic cosolvents, as in pure aqueous conditions, solvation of anions competes effectively with anion binding by receptor molecules. Here we address these challenges using a high-efficiency approach to discovery and implementation of chemosensors. We employ an automated synthesizer to assemble microbead-based chemosensors made from a large number of combinations of DNA-like building blocks, and we employ pattern-based recognition^8^ of fluorescence responses to differentiate anions without explicit design of receptor-binding chemistry. Our data document the ability of an 8-chemosensor set to discriminate all seventeen of these anion contaminants, some of which have not been the subjects of chemosensing before. Moreover, we find that the method can be quantitative for determining anion concentrations.

In this work, we set out to differentiate seventeen potential anion pollutants in aqueous media. We chose anions that range widely in elemental composition, oxidation states, and we included both organic and inorganic species commonly found in contaminated waters. They are as follows (with abbreviations/formulae): acetate (ac), arsenate (AsO_4_^3–^), azide (N_3_^–^), borate (H_2_BO_3_^–^), bromate (BrO_4_^–^), chromate (CrO_4_^2–^), cyanide (CN^–^), fluoride (F^–^), hypochlorite (ClO^–^), nitrate (NO_3_^–^), nitrite (NO_2_^–^), permanganate (MnO_4_^–^), phosphate dibasic (HPO_4_^2–^), oxalate (oxa), perchlorate (ClO_4_^–^), thiocyanate (SCN^–^), and selenate (SeO_4_^2–^). EPA limits for the toxic anions are listed in the ESI (Table S1†).

**Fig. 1:**
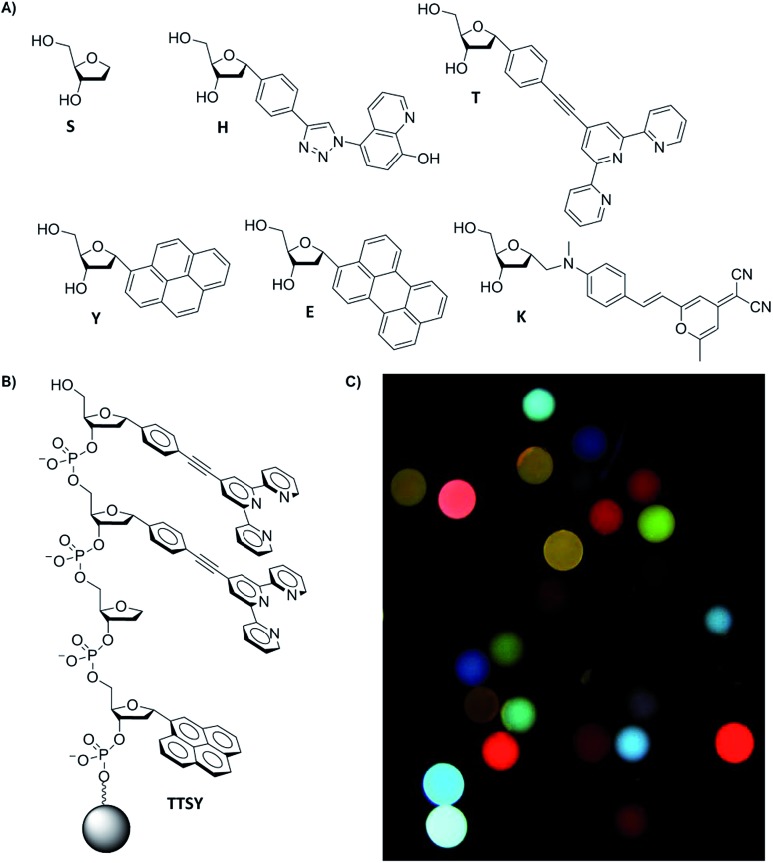
(a) Monomers included in the ODF library for anion detection. (b) An example of a tetrameric-length ODF sequence (TTSY, listed in 5′ → 3′) covalently attached to polyethylene glycol-polystyrene (PS) bead (130 μM). One bead contains many copies of one sequence, and the library is composed of 1296 unique sequences. (c) Sample image of ODF library captured by epifluorescence microscopy (*λ*_ex_ = 340–380 nm; *λ*_em_ > 420 nm) after incubation in 1 mM Tris–HCl buffer pH 8.

Our design strategy employed a set of DNA-like oligomeric compounds in which fluorophores replace natural nucleobases; these are termed oligodeoxyfluorosides or ODFs (Fig. 1). We have previously demonstrated cross-reactive recognition of analytes with sets of ODFs designed for interacting with vapors,^9^ metal cations,^10^ and bacterial metabolites.^11^ Anions might be expected to interact poorly in water with the polyanionic structure of DNA; to address this, we incorporated metal ligands into the ODF structure and pre-bound metals to the chemosensors prior to our sensing experiments. We retained the DNA backbone structure because the iterative synthesis enables ready assembly of a library of thousands of sequences by automated DNA synthesis. We employed a bead-based approach because it facilitates screening to identify multiple sensors with desirable fluorescence responses, and because ultimate implementation uses only a single bead per measurement, resulting in very low cost of materials. Further benefits of the ODF library include the ability to use a single excitation wavelength band (340–380 nm) for fluorescence analysis of all ODFs, and the stacked oligomeric structure encourages multiple forms of electronic interactions between the closely-spaced fluorophores,^12^ creating diverse emission responses from a relatively small number of monomers.

## Results and discussion

As mentioned above, we incorporated into our ODF oligomers two explicit fluorescent metal ligands (see monomers H and T, Fig. 1), which contain hydroxyquinoline and terpyridine ligands respectively. Monomer H was reported recently,^10*b*^ and monomer T was prepared for this work; it was chosen to add the ability to coordinate to varied transition metals and lanthanides, which in presence of anions can yield changes in fluorescence.^13,14^ We envisioned that the presence of terpyridine–metal complexes (and H–metal complexes as well) in the oligomeric structure might increase anion affinity, and binding to such metal sites might induce changes in fluorescence of the assemblies containing them. The metal ligands are flat, and thus may provide favorable stacking interaction with nearby fluorophores on the DNA backbone, and yet they retain space for anions to interact axially. Terpy-substituted DNAs have been reported previously,^15*a*^ but the structure of monomer T is new; it was synthesized from a brominated terpyridine intermediate and 4-α-phenylethynyl-substituted deoxyribose derivative; see Scheme S1 in the ESI† for details and characterization. Prior to its incorporation into the library, we measured the photophysical properties of the terpyridine monomer T. The Stokes shift of emission was relatively large: *λ*_abs,max_ = 293 nm, *λ*_em,max_ = 393 nm, and molar absorptivity was substantial, at 27 900 M^–1^ cm^–1^. Although T alone displayed a moderately low fluorescence quantum yield (*Φ*_fl_ = 5.7 ± 0.4% in 1 : 99 DMSO : H_2_O), its emission changed markedly in the presence of certain metals (Fig. S1†).

As metal cations to aid sensing, three lanthanides (Eu^III^, Tb^III^, Y^III^) and one transition metal (Zn^II^) were chosen for preincubation with our ligand-containing library. Recent examples of coordinated complexes of Eu^III^, Tb^III^, and Zn^II^ as anion sensors are well-documented.^5,13,15b^ Our studies of the monomer T alone in 1 : 99 DMSO : acetonitrile solution revealed large changes in absorption and in fluorescence emission due to apparent coordination with these metals (Fig. S2†).

With the metal-binding monomers in hand, next we constructed an ODF library of all possible tetramers containing six monomers (Fig. 1) (1296 unique sequences, named using letters of each monomer in 5′ → 3′ direction, Fig. 1b). Library members were covalently synthesized directly on 130 μm poly(ethylene glycol)-polystyrene beads using standard split-and-pool techniques.^12^ Pyrene (Y),^16^ perylene (E),^12^ and styrylpyran dye (K)^17^ nucleoside monomers serve as fluorescent components, an abasic monomer (S, Glen Research) was included as a spacer, and 8-hydroxyquinoline (H) and 2,2′;6′,2′′-terpyridine (T) monomers were included as possible fluorescent ligands for metals (Fig. 1a). The monomers are all *C*-glycosides (with α-anomeric centers) to avoid lability that can occur with C–N glycosidic bonds in the presence of metals.^18^ These monomers were derivatized as dimethoxytrityl (DMT)-protected phosphoramidites for library assembly by the standard oligonucleotide synthesis. The library was chemically tagged for decoding by methods of Still *et al.* ^19^ Details of library synthesis are provided in the ESI.† Epifluorescence microscopy images of a portion of the library revealed a wide range of emission wavelengths and brightness from varied combinations of the six monomers (Fig. 1c).

We proceeded to carry out screening to identify strongly-responding candidate ODF chemosensors for the varied anions in water. To achieve this, small portions of the library (*ca.* 50 beads at a time) were first preincubated with a metal cation (Eu^III^, Tb^III^, Y^III^, or Zn^II^) in acetonitrile (25 mM as nitrate salt hydrates) for thirty minutes and then thoroughly washed with water. The library members were then incubated with 1 mM Tris–HCl buffer (pH 8) for one hour to establish a background, imaged, and then tested with the seventeen anions at 250 μM. Comparisons of fluorescence images before anion/after anion were used to identify beads with the most pronounced fluorescence changes after thirty-minute anion exposure (Fig. S3†). Twenty-nine metal-bound candidate sensors were identified (see Table S2† for the list), and their sequences were resynthesized simultaneously on PS beads and on cleavable controlled pore glass (CPG). The cleaved ODFs were purified by HPLC and characterized (Table S2, Fig. S3 and S4†). The corresponding ODFs on beads were used in the following chemosensor experiments.

Having a set of strong responders to individual anions, we then set out to narrow the set to those with the strongest discriminating ability. Cross-testing the 29 candidate sensors with the 17 anions at 250 μM was carried out (same condition as screening; see ESI†), and statistical methods were used to identify the most diverse responders. To do this, numeric values of emission changes were determined from digital RGBL (red, green, blue, and luminosity) values of microscopy images extracted from 15 × 15 pixel squares at the center of beads (four repeats for each). Discriminant analysis (DA) and agglomerative hierarchical clustering (AHC) analysis of the cross-sensing data showed full discrimination of all 17 anions with the full set of sensors (Fig. S6 and S7†). The fluorescence color changes were relatively small, being captured by the digital analysis but not by strong visually observed changes, except for MnO_4_^–^ and CrO_4_^2–^ anions, which strongly quenched all chemosensors at the concentration tested.

Evaluation of some trends in the full cross-screening data was instructive. Most sensors containing monomer K (HYKY–Tb^III^, STKH–Tb^III^, SHKY–Y^III^, SKST–Y^III^, STKY–Y^III^, SYKY–Y^III^, SEKS–Zn^II^, and TTKS–Zn^II^) behaved with one general trend (increase in ΔR) with minor magnitude differences to the fifteen non-quenching anions. However, each of the sequences displayed subtle patterning differences (a “fingerprint”) which aided in the differentiation by pattern recognition. In another observation, we noted that sequence SYHS bound to Y^III^ and Zn^II^ showed remarkably different responses for the anion series, supporting the importance of metal cations in aiding sensing. Pairs of similar sensors sharing the same metal, such as STTS–Zn^II^ and STTE–Zn^II^, SYTS–Y^III^ and SYTY–Y^III^, and SHSY–Zn^II^ and SYHS–Zn^II^, also showed drastically different cross-reactive behavior; for example, selective lighting-up and color change responses to different anions. This strongly suggests that small differences in composition or sequence can alter the cooperative electronic and photophysical interaction of fluorophores. Taken together, the data show clearly that both metal identity and fluorescent monomer composition and sequence are important to the fluorescence response of the ODFS to varied anions.

**Fig. 2:**
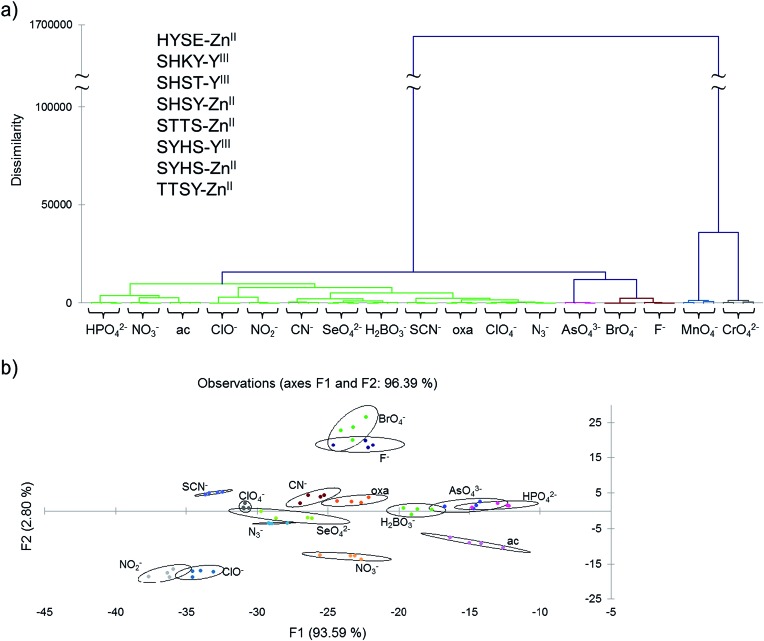
(a) Agglomerative hierarchial clustering (AHC) analysis showing the reduced eight-sensor set (see inset for list) achieving correct categorization of all seventeen anions at 250 μM in buffered water by measuring DRGB. Each ion was analyzed four times; in every case all four replicates were grouped together correctly. (b) 2-D discriminant analysis (DA) plot of the same data (chromate and permanganate were excluded for clarity as they were well separated from the rest). The two largest principal dimensions are shown. Ellipses represent 95% confidence levels around the centroids of four replicate points. See main text for explanation of the abbreviations.

Examination of the statistical data revealed that some sensors behaved similarly (for example, the chemical isomers HYES–Zn^II^ and HYSE–Zn^II^, Fig. S7†), which allowed us to remove much of the redundancy and overlap of these similarities by eliminating less-discriminating chemosensors from the analysis. By doing this, we were able to reduce the analysis to an eight-chemosensor set that showed complete discrimination of all seventeen anions based on AHC analysis (sequences are given in Fig. 2).

**Fig. 3:**
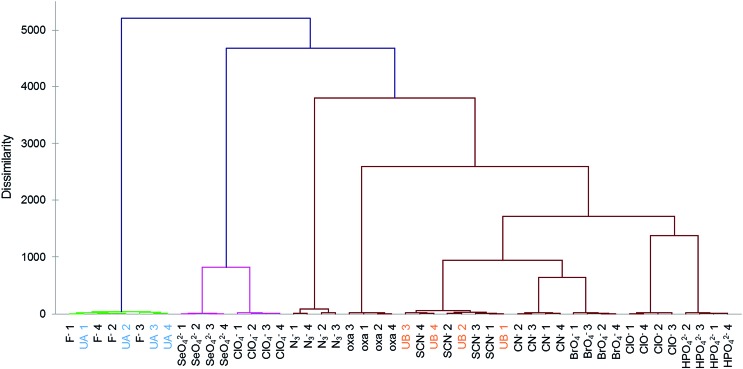
AHC analysis showed correct identification of two unknown anion samples (250 μM in buffered water) chosen blindly (UA = F^–^; UB = SCN^–^) using ten standards measured with eight-sensor set. The numbered data points indicate four replicates (four sensor beads) for each analyte.

To further test the effectiveness of this final 8-chemosensor set in pattern-based analysis, two anions were selected blindly out of ten anions that were close in responses; these were analyzed separately and the responses compared with the known data. We were able to correctly identify all four replicates of the unknown samples at 250 μM by matching them to their respective standards (Fig. 3 and S8a†), using agglomerative clustering. The unknowns proved to be fluoride and thiocyanate. Confusion matrix analysis also predicted correctly all twelve samples (Fig. S8b†).

**Fig. 4:**
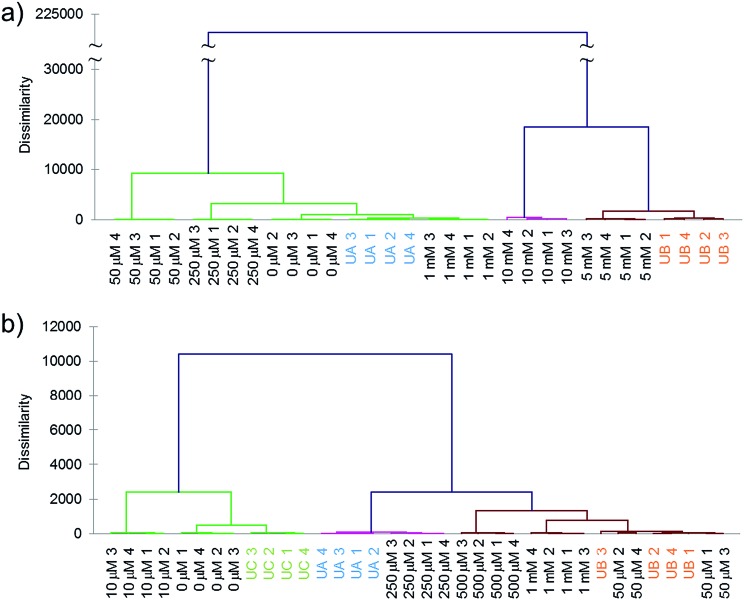
Quantification of unknown concentration of anion specimens by matching with standards, measured with eight-sensor ODF set. (a) Two samples of cyanide at unknown concentration between 0 and 10 mM in buffered water (UA = 1 mM; UB = 5 mM) were correctly grouped based on AHC analysis. (b) Two out of three unknown samples of selenate (UA = 250 μM; UB = 50 μM; UC = 10 μM) showed correct grouping using the same method. UA and UB matched the standard response but not for UC, indicative of a detection limit. The numbered data points indicate four replicates (four sensor beads).

Next we tested whether the eight-ODF set could be used to determine the concentration of a given anion by matching with standards. Blind concentration tests were performed for cyanide from 0 to 10 mM with two unknown samples: “UA” (1 mM) and “UB” (5 mM). For selenate, a range from 0 to 1 mM with three unknowns: “UA” (250 μM), “UB” (50 μM), and “UC” (10 μM) was tested. Based on AHC analysis, the eight sensor set was able to distinguish cyanide as low as 1 mM (Fig. 4a) and selenate as low as 50 μM (Fig. 4b). For both anions, some individual ODF sensors showed nonlinear response in the three color channels leading to ungraphable points as function of concentration in the two largest principal axes. For example, 1 mM cyanide elicited ΔRGBL responses more similar to the blank than 50 or 250 μM. This may be indicative of different anion recruitment processes at different concentrations. However, it was still possible to deduce the unknown concentrations by proximity of the centroids to the standards in DA plots (Fig. S9†).

**Fig. 5:**
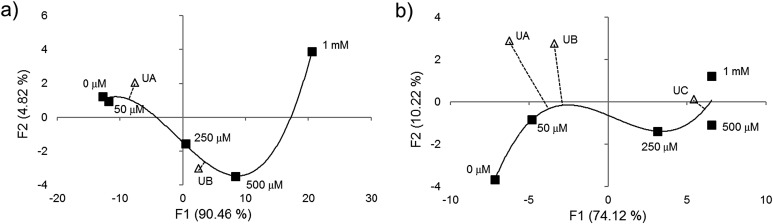
DA analysis of unknown concentration samples for (a) SCN^–^ and (b) AsO_4_^3–^ using calibration curves generated by standard concentrations noted on the figure. Smooth curves were plotted through calibration standards, and unknown responses were mapped onto the nearest points on the curves. Only sensors with increasing magnitudes of ΔRGB *versus* concentration were used (four out of eight for both anions; see main text for list). Centroids of four trials performed on separate occasions were plotted (black squares), and calibration curves were plotted using a best-fit curve. Unknown concentrations (white triangles representing centroid of four trials) were compared with interpolation using shortest orthogonal line to the curves. (a) UB (300 μM) was interpolated successfully but not UA (30 μM). (b) For arsenate, UC was successfully interpolated at 800 μM but not UA (5 μM) nor UB (20 μM), indicating a detection limit above these concentrations.

Encouraged by the initial quantification data, we carried out further quantification tests of other selected anions. For thiocyanate, two solutions were prepared with concentrations chosen randomly between 0 to 1 mM, and the four responses from the sensor set were measured on separate occasions to account for photobleaching and stability during the time course of the experiment. The standard deviations of ΔRGB were larger in general, with chemosensor output apparently fluctuating due to minor differences in the exposure to the microscope UV source and aging of the ODFs over the time course of experiment. Even so, four sensors (HYSE–Zn^II^, SHKY–Y^III^, STTS–Zn^II^, and SYHS–Y^III^) still showed increasing magnitudes of emission response with increasing concentration (Fig. S10†). The patterned response of these sensors was used to plot a calibration curve using two largest principal axes from DA. Fig. 5a shows the plot of centroids from the quadruplicates, and we were able to interpolate the unknown concentrations based on orthogonal lines from the calibration curve confidently for 300 μM (sample “UB”), but not for 30 μM (“UA”), indicative of the detection limit. This was comparable to other thiocyanate-specific fluorescent sensors which, unlike the current chemosensors, require organic cosolvents.^20^ In the current experiments, ODFs with nonzero responses showed complex behavior, which suggests the action of multiple interactive processes, which may be expected considering the polyfunctional design of the ODFs.

Similar testing was performed for three unknown concentrations of arsenate. A different set of four sensors (SHKY–Y^III^, SHST–Y^III^, STTS–Zn^II^, TTSY–Zn^II^) showed simple positive or negative correlation from 0 to 1 mM and their responses were used to plot the calibration curve (Fig. S10†). The unknown concentration of 800 μM, “UC”, was able to be interpolated but not for 5 μM (“UA”) or 20 μM (“UB”), thus we conclude that the detection limit for arsenate falls between 20 and 800 μM (Fig. 5b). Taken together, the data show that a set of as few as eight chemosensors on beads can reproducibly discriminate this large set of 17 toxic anions under fully aqueous conditions, and can operate quantitatively as well, with detection limits ranging from millimolar to low micromolar.

Overall, we have shown that the use of the DNA backbone to assemble fluorescent chemosensors on beads yields diverse and sensitive detection of a broad array of anions using a pattern-based analytical approach. Prior examples of pattern-based optical chemosensors for anions are relatively scarce in the literature, but comparisons are informative. Anslyn *et al.* ^8*b*^ described microbeads containing cationic amino acids and a combinatorial tripeptide arm for selective displacement of pre-bound fluorescein dye. Their 30-sensor array distinguished three biological phosphates at 20 mM. Anzenbacher *et al.* ^8*c*^ described a hydrogen-bonding array of seven calix[4]pyrrole analogues and an anthraquinone-based dipyrrolyquinoxaline in polyurethane hydrogel that was able to distinguish 10 anions at 5 mM and above in water; the greatest sensitivity was to fluoride and acetate. Another sensor array by the same laboratory employed a single calix[4]pyrrole in 10 different poly(ether-urethane) matrices of varying ratios.^8*d*^ This 10-component array was able to distinguish nine anions at 500 μM in water. In contrast to these studies, our current ODF sensor set achieved full differentiation of 17 anions at 250 μM in water using only eight sensor compounds. We hypothesize that the high dimensionality of optical response of the composite fluorophores in the ODF design yields improved analyte differentiating power, allowing the use of fewer chemosensors to distinguish a greater number of analytes.

By far the largest body of literature on anion optical sensing has focused on one-sensor, one-analyte molecular designs. Using this classical approach, fluorescence chemosensors and chemodosimeters of several of the anions targeted here have been described in the literature. Significant examples include chemodosimeters of ClO^–^, which could report on concentrations as low as 1 nM in the presence of organic cosolvents or 0.8 μM in a fully aqueous example.^21^ Recent examples of nitrite sensing also involve chemodosimeters, employed with organic cosolvent or acidic medium to achieve sensitivity of 2 mM to as low as 20 μM.^22^ Relatively few prior reports have described sensing of azide in water, but one significant study using a luminescent copper complex reported a detection limit of 6 μM.^23^ Few reports of perchlorate chemosensing exist in the literature, but one example described sensitivity of 100 mM.^14*b*^ Examples of fluorescent chemosensors of the oxyanion form of arsenic are rare; recent examples were carried out in partly organic solvent, but yielded sensitivity as low as 5 nM.^24^ Copper complexes have been employed successfully in the sensing of oxalate anion, yielding sensitivities in water to concentrations as low as 79 nM.^25^ A number of studies developing inorganic phosphate chemosensors exist, with detection limits ranging from low micromolar to 51 nM.^26^ Cyanide sensing has been the subject of many reports; significant examples of fully aqueous sensing have been demonstrated with detection limits down to low nanomolar concentrations.^27^ Finally, fluoride ion has also been a popular analyte for fluorescence chemosensing. While most recent examples of F^–^ sensing displayed detection limits in the micromolar range, some remarkable nanomolar (24 nM)^28*a*^ and sub-nanomolar (50 pM)^28*b*^ detection limits in water have been reported.

In comparison to those individual selective chemosensors, our ODF array displays less sensitivity to a number of these anions (AsO_4_^3–^, ClO^–^, CN^–^, F^–^, N_3_^–^, NO_2_^–^, CrO_4_^2–^, MnO_4_^–^, NO_3_^–^, acetate and oxalate) than some of the most sensitive examples in the literature, although it is worth noting that we have restricted our experiments to purely aqueous sensing, which has proven more difficult for many small-molecule organic chemosensors in the past. For thiocyanate, our ODF set exhibits sensitivity approximately equivalent to literature values. In addition, for at least one case (ClO_4_^–^) our ODFs display considerably better sensitivity than prior reports. Moreover, we have shown that the ODF fluorescent chemosensors can report on some toxic anions for which little or no precedent exists (*e.g.*, borate, BrO_4_^–^, and SeO_4_^2–^). Thus the versatility of the current molecular chemosensing approach is noteworthy, and illustrates the value of pattern-based detection with optically diverse molecules. It is worth pointing out, in this regard, that the sensitivity of the current ODF set is significantly better than the prior pattern-based anion sensing arrays reported recently, and as noted above, the discriminating potential with a greater number of anions has been demonstrated here.

The current chemosensing design is promising in a number of respects for practical application in toxic anion analysis. The amount of material used in a measurement is very low (*ca.* 30 picomoles on a bead), and so cost per experiment is very little. This is in contrast with standard small-molecule sensing, which is most often carried out in solution and thus requires (typically) micromolar concentrations of a chemosensor compound. Synthesis of the current chemosensor ODFs on beads is done in automated fashion in minutes on a DNA synthesizer, and different sequences are merely programmed from a small set of monomers. A single synthesis (1 mmol standard scale) produces *ca.* 32 000 beads of a chemosensor. The volume of sample required is also small, with use of as little as 50 microliters being feasible, and measurements are completed in 30 minutes. Requiring only three color channels acquired from microscopy or other miniaturized RGB camera imaging, this approach may hold promise for on-site monitoring, assuming a method for immobilizing beads or otherwise arraying ODFs can be developed.

It is interesting to speculate on the origins of fluorescence responses in this library. A large portion of the screened sensors contained S at the 5′ end of the sequence, perhaps due to lack of nucleobase freeing up space for metal cations and anion recruitment. Y^III^ and Zn^II^ were predominant in strong responders, and while coordinated Zn^II^ is well-established for anion sensing,^5^ examples of the use of Y^III^ in anion sensing are rare, if any, in the literature. It is interesting to note that among the screened ODF sequences, SYHS were chosen by screening for two different anions and each complexed to Y^III^ (cyan) and Zn^II^ (green), respectively, with different emission colors (Fig. S11†). A number of ODFs with only minor differences in composition or sequence were also selected, further demonstrating varied sensing behavior from a small number of monomer combinations. Fluorescence emission colors of these ODF beads changed after exposure to metals, as seen from microscopy images (Fig. S11†). Whether the color changes signify coordination of metals by ODF or other unknown processes (such as adsorption into the polystyrene matrix) is not yet determined. Interestingly, several sequences were identified in sensing responses (*e.g.*, SEKS, SSSE, and SYKY) that do not contain any apparent metal ligands but nevertheless showed emission changes after introduction of metals; this suggests that other metal recruitment mechanisms are available beyond the obvious binding to terpyridine or hydroxyquinoline ligands. Future experiments will address mechanisms of anion sensing in more detail.

## Conclusions

In summary, we have developed a small set of chemosensors built on DNA backbone with the ability to readily differentiate seventeen anion pollutants in aqueous media at micromolar concentrations. The sensing experiments required only a thirty minute exposure to the analyte of interest and were accomplished on microscopic scale with purely optical imaging. Beyond the qualitative discrimination of known samples, an eight-sensor subset also succeeded in identifying unknown solutions of fluoride and thiocyanate out of ten closely responding anions using agglomerative hierarchical clustering, and demonstrated successful quantification using pattern recognition.
