# Correction: Pattern-based detection of anion pollutants in water with DNA polyfluorophores

**DOI:** 10.1039/c5sc90036k

**Published:** 2015-06-24

**Authors:** Hyukin Kwon, Wei Jiang, Eric T. Kool

**Affiliations:** a Department of Chemistry , Stanford University , Stanford , California 94305-5080 , USA . Email: kool@stanford.edu ; Fax: +1 650 725 0259 ; Tel: +1 650 724 4741

## Abstract

Correction for ‘Pattern-based detection of anion pollutants in water with DNA polyfluorophores’ by Hyukin Kwon *et al.*, *Chem. Sci.*, 2015, **6**, 2575–2583.



## 

On page 2581, an NIH grant was inadvertently omitted in the Acknowledgements. The Acknowledgements should read as follows:

We thank Eni S.p.A. and the U.S. National Institutes of Health (GM106067) for support.

The Royal Society of Chemistry apologises for these errors and any consequent inconvenience to authors and readers.

