# Supplementary material for: Pattern-based detection of anion pollutants in water with DNA polyfluorophores
Source: Chem Sci. 2015 Feb 18;6(4):2575–83. doi: 10.1039/c4sc03992k (PMC4486361; doi:10.1039/c4sc03992k)
Supplement: Supplementary file 1 [file SC-006-C4SC03992K-s001.pdf]

Electronic Supplementary Material (ESI) for Chemical Science.
This journal is © The Royal Society of Chemistry 2015

Supporting Information

Toxic Anion Sensing with DNA: Pattern-Based Detection of Anion Pollutants in Water with DNA Polyfluorophores

Hyukin Kwon, Wei Jiang, and Eric T. Kool*

Department of Chemistry, Stanford University, Stanford, CA 94305 (USA); E-mail: kool@stanford.edu

Table of Contents

Experimental Methods (p. 2)
Table S1. United States EPA limits for toxic anions in drinking water. (p. 5)
Scheme 1. Synthesis of ODF T monomer (**4**) and derivatization for automated DNA synthesis. (p. 6)
Figure S1. Digital photo of 1 mL of T monomer with varied metal nitrate salts . (p. 9)
Figure S2. Absorption and emission spectra of T monomer (p. 9)
Figure S3. Example of ODF anion sensor screening. (p. 10)
Table S2. MALDI-TOF mass spectrometry of the resynthesized ODF sequences. (p. 11)
Figure S4. Absorption spectra of resynthesized ODF sequences (20 μM) in PBS buffer. (p. 12)
Figure S5. Fluorescence emission spectra of resynthesized ODF sequences. (p. 13)
Figure S6. Discriminant analysis (DA) and agglomerative hierarchial clustering (AHC) of 29 screened sensor set with seventeen anions at 250 μM (p. 14)
Figure S7. Quantitative fluorescence responses of 29 ODF sensors to anions. (p. 16)
Figure S8. Unknown anion identification test using the eight-sensor set. (p. 17)
Figure S9. DA plots of cyanide (left) and selenate (right) with unknown concentrations. (p. 17)
Figure S10. Fluorescence responses from unknown concentrations of SCN^- and AsO_4^{3-} . (p. 18)
Figure S11. Sample images of twenty-nine resynthesized ODF tetramers on PS beads. (p. 19)
References (p. 20)

Experimental Methods

Chemicals and reagents

Anhydrous solvents were purchased from Acros Organics and used without further purification. Chemicals for oligodeoxyfluoroside (ODF) monomer synthesis, anion sodium salts, and metal nitrates were purchased from Sigma-Aldrich unless otherwise noted. All anion solutions were prepared within hours of use. Chemicals used for solid-phase synthesis of ODF were purchased from Glen Research, including spacer (S) phosphoramidites, 3'-phosphate CPG, synthesizer reagent solutions and deprotection reagent. All chemical reactions were performed under argon gas unless otherwise noted. Silica gel (60 Å, 200-425 mesh) was used for flash column chromatography.

General instrumentation.

^1H, ^{13}C, and ^{31}P NMR spectra were recorded using Varian Innova 400 MHz instrument unless otherwise noted. Internal signal from NMR solvents ($CDCl_3$ or DMSO-d_6) were used as references. Chemical shifts are reported as ppm, and multiplicity patterns are abbreviated as the following: singlet (s), doublet (d), triplet (t), and multiplet (m). Mass spectra were obtained using ESI or MALDI-TOF at Stanford University Mass Spectrometry Facility and Stanford Protein and Nucleic Acid Facility, respectively. Gas chromatography was performed using Shimazu GC17A instrument (EC detector). HPLC was performed using Shimazu LC-20AD (SPD-M20A diode array detector) and reverse phase C5 column (Phenomenex Jupiter). Absorption spectra were obtained using Varian Cary 100 Bio UV-Vis Spectrophotometer. Steady-state fluorescence emission spectra were measured on Jobin Yvon-Spex Fluorolog 3 spectrometer.

ODF library construction.

The library was assembled on amine-functionalized polyethylene glycol-polystyrene beads (PS beads, 130 μm, NovaSyn TG amino resin) as previously reported[1] to yield 1296 unique sequences of tetramers. Binary chemical tags[2] were installed during the library synthesis and later cleaved for gas chromatographic sequence identification (see below). ODFs were deprotected using 50 mM potassium carbonate in methanol, washed with EDTA in dimethylformamide (DMF), water, acetonitrile, and lastly with dichloromethane (DCM), and dried using argon stream.

Library screening.

About fifty beads from the library were pre-equilibrated with one of the four metals: $Eu(NO_3)_3{\cdot}5H_2O$, $Tb(NO_3)_3{\cdot}5H_2O$, $Y(NO_3)_3{\cdot}4\ H_2O$, and $(Zn(NO_3)_2{\cdot}6H_2O$. The beads were shaken in 1 mL of 25 mM metal solution in acetonitrile for 30 minutes and then thoroughly washed with water and acetonitrile. They were placed on a small square removable double-sided tape (5 mm, 3M Scotch) attached to a Petri dish (35 mm diameter, Falcon). 25 μL of 1 mM Tris-HCl buffer (pH 8) was added and let soak for one

hour at room temperature protected from light. A digital image was captured under epifluorescence microscope (Nikon Eclipse 80i, equipped with Nikon Plan Fluor 4x/0.13 objective, ND8 filter, and QIClick digitial CCD camera) using λ_{ex} = 340-380 nm and λ_{em} > 420 nm filters. The exposure times were set constant during screening (80 ms for each RGB channel, gain 4x, 24-bit image). Next, anion (500 μM) in the same buffer (25 μL) was added to above and incubated for 30 minutes at room temperature. A second fluorescence image was captured using the same microscope setting. Comparing the "before" and "after" picture visually, noticeable and desirable fluorescence color changes were noted and these beads were isolated. They were placed in a sealed capillary tube, and the chemical tags were released using 3 μL of CAN solution (0.5 M ceric ammonium nitrate in 1:1 water:acetonitrile) and 3 μL of decane. The capillary tubes were sonicated for three hours, centrifuged briefly, and the organic layer was derivatized with *N*,*O*-Bis(trimethylsilyl)acetamide and analyzed with gas chromatography to decode the sequence.

Resynthesis and characterization of screened ODF sensors.

The screened ODF sequences were resynthesized on ABI 394 DNA synthesizer using standard phosphoramidite oligonucleotide synthesis. Both 3'-phosphate CPG (1 μmol, Glen Research) and 10 mg dimethoxytrityl (DMT)-functionalized PS beads (0.29 mmol/g) were added to DNA synthesis columns to allow simultaneous synthesis of both cleavable and solid-phase ODF sequences. The two solid supports were separated after synthesis in DCM, and each was deprotected with 50 mM potassium carbonate in methanol to afford cleaved ODF sequence in solution and ready-to-use ODF on PS beads. The solid-phase ODFs were washed with water and acetonitrile and dried with argon before use. Cleaved ODFs were filtered, dried, and purified by HPLC using C5 reverse-phase column and 50 mM triethylammonium acetate and acetonitrile as mobile phase. The purified sequences were redissolved in 0.5 mL water and characterized by MALDI-TOF mass spectrometry (Table S2), and its optical properties (absorption and fluorescence emission spectra, Figure S4 and S5) were measured in phosphate-buffered saline (PBS).

Resynthesized sensor anion cross-screening.

Resynthesized ODF sensors on PS beads pre-equilibrated with one of the four metals (see library screening section for details) and placed in a Petri dish (35mm diameter) using a small square piece (5 mm) of removable double-sided tape. They were incubated in 3 mL of 1 mM Tris-HCl buffer (pH 8) for one hour at room temperature protected from light, and the "before" image was taken under epifluorescence microscope using λ_{ex} = 340-380 nm and λ_{em} > 420 nm filters (see above). The exposure times were constant for each sequence throughout the experiments and ranged from 50 ms to 200 ms (for all RGB channels, gain 4x) to avoid over-exposure. The solution was then replaced with 3 mL of the same buffer containing the anion of interest at 250 μM, and the "after" image was captured after 30 minutes. Four beads were randomly chosen and a 15-by-15 pixel square was placed at the center of each bead in Adobe Photoshop CS5. Mean RGBL values over the pixel selection were extracted and the difference values (ΔR, ΔG, ΔB, and ΔL, theoretical range from -255 to +255) were obtained with error indicating standard deviation of the four beads. For unknown concentration tests of SCN^- and AsO_4^{3-}, one bead was

randomly selected for processing using the same method, and each sample was tested at four different occasions.

Statistical methods.

ΔR, ΔG, and ΔB from each bead were used for discriminant analysis (DA) and agglomerative hierarchical clustering (AHC). Addinsoft XLSTAT was used to generate both analyses. For DA, ellipses around the centroid represent 95% confidence limit. For AHC, dissimilarity was analyzed on Euclidean distance using Ward's agglormeration method.

Table S1. United States Environmental Protection Agency limits for toxic anions in drinking water.

Anion	Abbreviation	MCL[a] (mg/L)
acetate	ac	N/A
arsenate	AsO_4^{3-}	0.010 as arsenic
azide	N_3^-	N/A
borate	$H_2BO_3^-$	N/A
bromate	BrO_4^-	0.010
chromate	CrO_4^{2-}	0.1 as chromium
cyanide	CN^-	0.2
fluoride	F^-	4.0
hypochlorite	ClO^-	1.0 as chlorite
nitrate	$NO3^-$	10
nitrite	$NO2^-$	1
permanganate	MnO_4^-	N/A
phosphate dibasic	HPO_4^{2-}	N/A
oxalate	oxa	N/A
perchlorate	ClO_4^-	N/A
thiocyanate	SCN^-	N/A
selenate	SeO_4^{2-}	0.05 as selenium

[a] Maximum contaminant level for primary drinking water regulations.

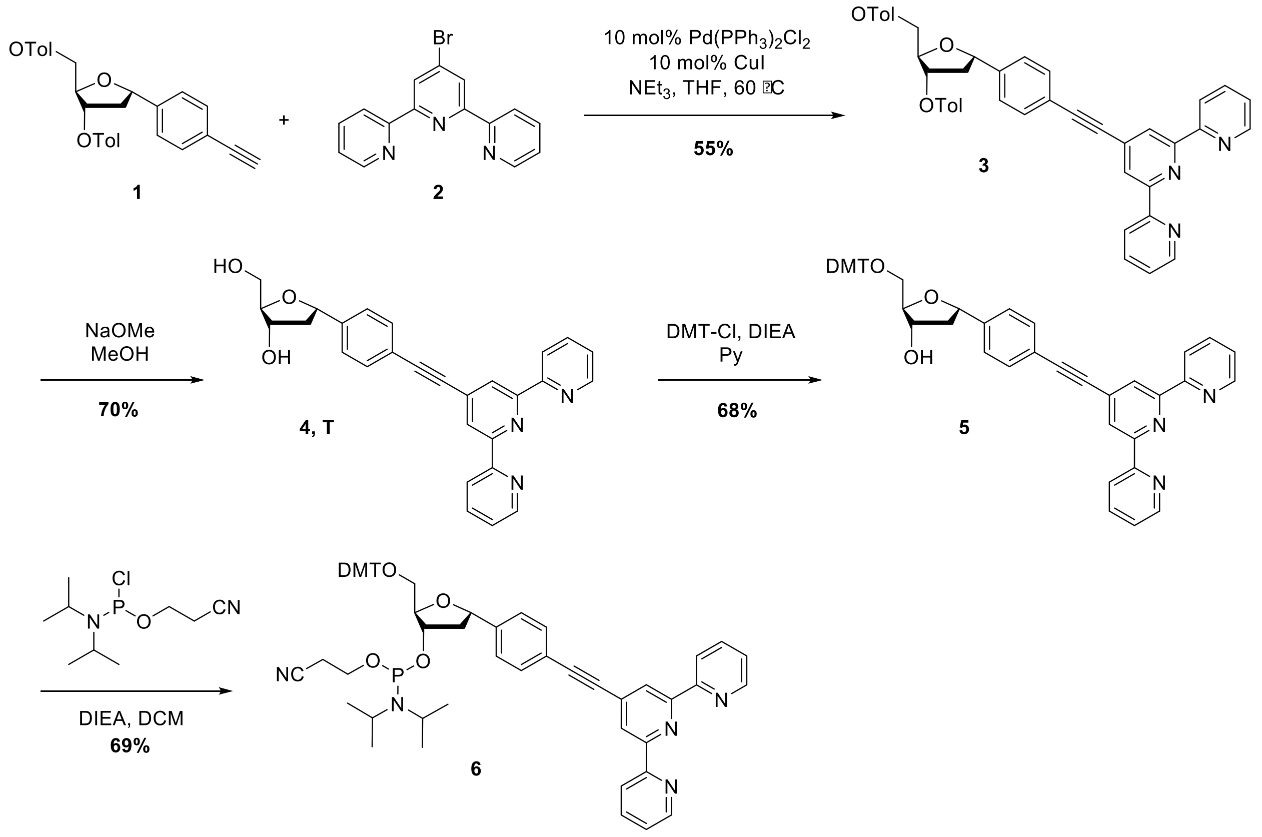

Scheme 1. Synthesis of ODF T monomer (**4**) and derivatization for automated DNA synthesis.

(2R,3S,5S)-5-(4-([2,2':6',2''-terpyridin]-4'-ylethynyl)phenyl)-2-(((4-methylbenzoyl)oxy)methyl)tetrahydrofuran-3-yl 4-methylbenzoate (3)

Bis-toluoyl-protected 4-phenylethynyldeoxyribose (**1**, 0.28 g, 0.62 mmol)[3] was dissolved in 10 mL anhydrous tetrahydrofuran (THF). 4'-Bromo-2,2':6',2''-terpyridine (**2**, 0.16 g, 0.513 mmol, TCI America) was added to above followed by $Pd(PPh_3)_2Cl_2$ (36 mg, 0.062 mmol), CuI (10 mg, 0.062 mmol), and triethylamine (5 mL). The reaction was stirred vigorously, and the mixture turned dark black within minutes. It was allowed to stir at 60 °C for 5 h then cooled to room temperature. The solvents were evaporated under vacuum and the crude (black oil) was loaded directly onto flash column chromatography (gradient from 10:1 hexanes:ethyl acetate to 2:1). The product **3** was isolated as yellow foam (0.23 g, 55%). ^{1}H NMR ($CDCl_3$, δ): 8.71 – 8.68 (m, 2H), 8.62 – 8.57 (m, 4H), 7.97 (d, J = 8.2 Hz, 2H), 7.84 (td, J = 7.7, 1.8 Hz, 2H), 7.63 (d, J = 8.2 Hz, 2H), 7.57 (d, J = 8.3 Hz, 2H), 7.45 (d, J = 8.1 Hz, 2H), 7.32 (ddd, J = 7.5, 4.8, 1.1 Hz, 2H), 7.23 (d, J = 8.0 Hz, 2H), 7.17 (d, J = 8.0 Hz, 2H), 5.60 (dt, J = 6.2, 3.0 Hz, 1H), 5.42 (dd, J = 7.6, 5.2 Hz, 1H), 4.73 – 4.67 (m, 1H), 4.58 (dd, J = 4.7, 1.6 Hz, 2H), 2.98 – 2.89 (m, 1H), 2.39 (s, 3H), 2.36 (s, 3H), 2.34 – 2.30 (m, 1H). ^{13}C NMR ($CDCl_3$, δ): 166.33, 165.99, 155.56, 155.46, 149.13, 144.08, 143.89, 143.86, 136.89, 133.34, 132.02, 129.71, 129.58, 129.16, 129.08, 127.01, 126.65, 125.65, 124.01, 122.74, 121.31, 121.17, 93.65, 87.48, 82.49, 79.91, 76.30, 64.57, 40.2, 21.67, 21.65. HRMS (ESI, m/z): $[M+H]^+$ calculated for $C_{44}H_{36}N_3O_5$: 686.2649; found: 686.2642.

(2R,3S,5S)-5-(4-([2,2':6',2''-terpyridin]-4'-ylethynyl)phenyl)-2-(hydroxymethyl)tetrahydrofuran-3-ol (4, T monomer)

3 (2.07 g, 3.02 mmol) was dissolved in 70 mL DCM (clear brown solution). Sodium methoxide in methanol (25% wt, 2.07 mL) was added dropwise and stirred overnight (brown precipitate mixture). The solvents were evaporated under vacuum and the crude (brown oil) was purified by flash column chromatography (6 to 10% methanol in DCM with 15% pyridine as cosolvent). The product **4** was washed with chloroform and then methanol (yellow solid, 0.95 g, 70%). ^{1}H NMR (DMSO-d_6, δ): 8.73 (d, J = 4.4 Hz, 2H), 8.62 (d, J = 7.9 Hz, 2H), 8.47 (s, 2H), 8.03 (t, J = 7.7 Hz, 2H), 7.66 (d, J = 8.0 Hz, 2H), 7.57 – 7.50 (m, 2H), 7.47 (d, J = 8.1 Hz, 2H), 5.02 (t, J = 7.6 Hz, 1H), 4.23 (dd, J = 11.8, 6.3 Hz, 1H), 3.89 (dd, J = 9.2, 4.7 Hz, 1H), 3.56 (dd, J = 11.6, 3.8 Hz, 1H), 3.47 (dd, J = 11.7, 5.2 Hz, 1H), 2.66 – 2.56 (m, 1H), 1.80 – 1.69 (m, 1H). ^{13}C NMR (DMSO-d_6, δ): 155.26, 154.14, 149.34, 146.00, 137.65, 132.54, 131.86, 126.21, 124.83, 121.88, 120.92, 119.66, 94.08, 86.72, 86.60, 78.26, 71.68, 61.70, 43.38. HRMS (ESI, m/z): $[M+H]^+$ calculated for $C_{28}H_{24}N_3O_3$: 450.1812; found: 450.1806.

(2R,3S,5S)-5-(4-([2,2':6',2''-terpyridin]-4'-ylethynyl)phenyl)-2-((bis(4-methoxyphenyl)(phenyl)methoxy)methyl)tetrahydrofuran-3-ol (5)

4 (0.50 g, 1.11 mmol) was coevaporated with 10 mL anhydrous pyridine twice and then dissolved in 30 mL anhydrous pyridine (yellow solution). *N,N*-Diisopropylethylamine (DIEA, 0.58 mL, 3.33 mmol) was added to above, and DMT-Cl (1.13 g, 3.33 mmol) was added in several portions to the stirring mixture (brown solution). The reaction was stirred for 3 h at room temperature after which thin layer chromatography (TLC) showed absence of the starting material. Methanol (1 mL) was added, and the solvents were evaporated under vacuum. The crude brown oil was purified by flash column chromatography (from 2:1 hexanes:ethyl acetate to 1:1, then ethyl acetate; all mobile phase included 3% triethylamine as cosolvent) to yield the product as light yellow foam **5** (0.57 g, 68%). ^{1}H NMR (DMSO-d_6, δ): 8.66 (ddd, J = 4.8, 1.8, 0.9 Hz, 2H), 8.60 – 8.54 (m, 4H), 7.85 – 7.79 (m, 2H), 7.54 (d, J = 8.4 Hz, 2H), 7.48 (dd, J = 8.4, 1.2 Hz, 2H), 7.42 (d, J = 8.1 Hz, 2H), 7.39 – 7.34 (m, 4H), 7.32 – 7.26 (m, 4H), 7.21 (dt, J = 9.3, 4.2 Hz, 2H), 6.85 – 6.79 (m, 4H), 5.16 (t, J = 7.5 Hz, 1H), 4.50 (dd, J = 11.3, 6.5 Hz, 1H), 4.25 (q, J = 4.8 Hz, 1H), 3.75 (d, J = 0.5 Hz, 6H), 3.31 (ddd, J = 18.7, 9.7, 4.9 Hz, 3H), 2.73 (dt, J = 13.3, 6.8 Hz, 1H), 2.05 (m, 1H). ^{13}C NMR ($CDCl_3$, δ): 158.70, 155.79, 155.67, 149.36, 145.16, 144.71, 137.23, 136.28, 136.26, 133.68, 132.33, 130.34, 130.32, 128.41, 128.12, 127.05, 126.23, 124.32, 123.07, 121.56, 121.49, 113.40, 94.22, 87.64, 86.50, 85.16, 79.75, 74.61, 64.83, 60.69, 55.43, 43.66, 21.31, 14.46. HRMS (ESI, m/z): $[M+H]^+$ calculated for $C_{49}H_{42}N_3O_5$: 752.3119; found: 752.3126.

(2R,3S,5S)-5-(4-([2,2':6',2''-terpyridin]-4'-ylethynyl)phenyl)-2-((bis(4-methoxyphenyl)(phenyl)-methoxy)methyl)tetrahydrofuran-3-yl (2-cyanoethyl) diisopropylphosphoramidite (6)

5 (0.090 g, 0.12 mmol) was dissolved in 5 mL anhydrous DCM. DIEA (0.13 mL, 0.72 mmol) was added to above, and 2-cyanoethyl *N,N*-diisopropylchlorophosphoramidite (0.080 mL, 0.36 mmol) was added dropwise. The reaction was allowed to stir overnight at room temperature. The solvents were evaporated under vacuum and the crude (clear oil) was quickly purified using flash column chromatography (4:1 hexanes:ethyl acetate to 2:1; all mobile phase included 4% triethylamine) to yield the product **6** as white foam (0.10 g, 69%). **6** quickly oxidized in organic solvents and was immediately used for library synthesis or ODF sensor resynthesis without further characterization. ^{31}P NMR ($CDCl_3$, δ): 149.32, 148.59.

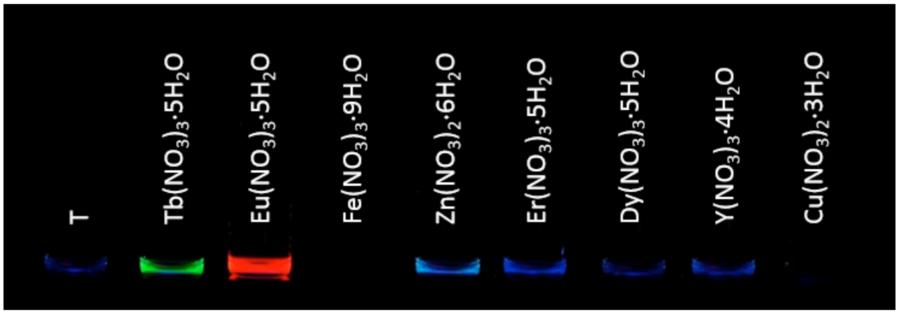

Figure S1. Digital photo of 1 mL of T monomer (200 μM in 1:4 DMSO:CH_3CN, λ_{ex} = 365 nm) in a glass vial (far left) and after addition of varied metal nitrate salts (20 mM, 10 min equilibration).

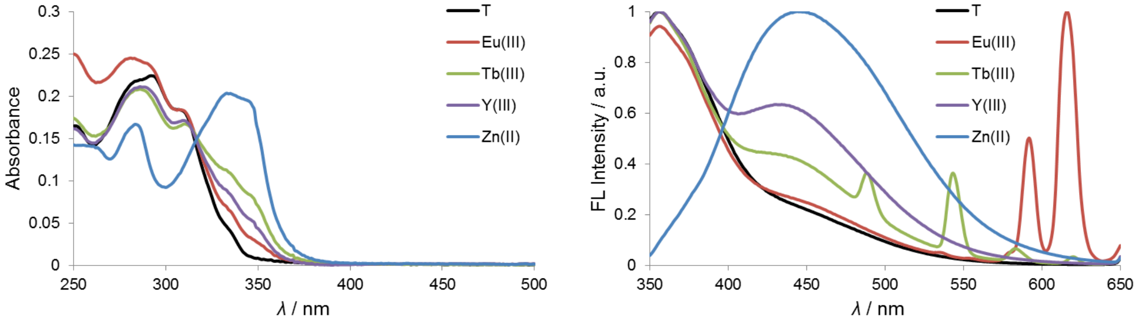

Figure S2. Absorption (left) and fluorescence emission (right, λ_{ex} = 330 nm) spectra of T monomer (5 μM) and after addition of metal nitrate salts in 1:99 DMSO:acetonitrile solution (5 eq, after ten minutes).

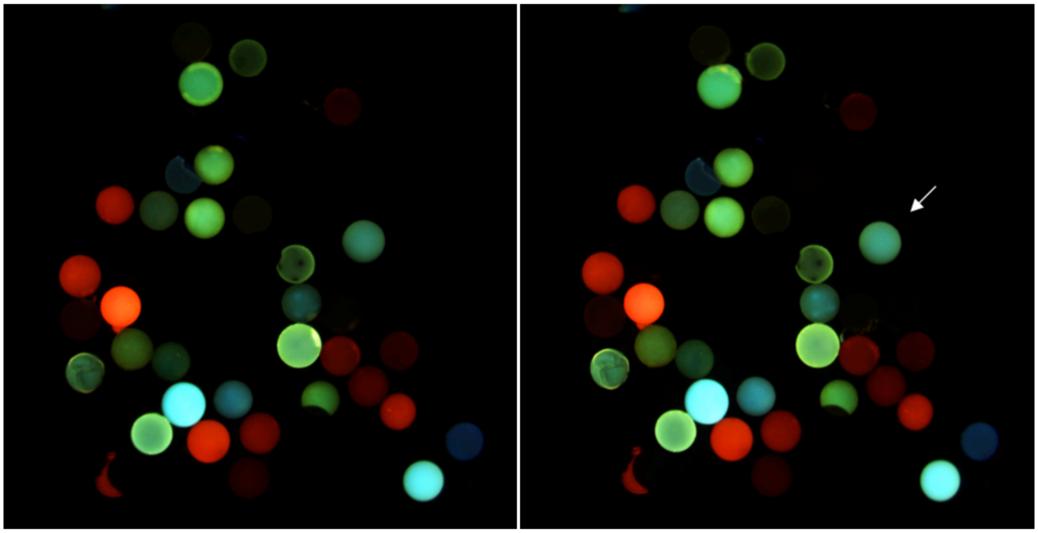

Figure S3. Example of ODF anion sensor screening. A batch of ODF library on beads was preincubated with Zn^{II} and then incubated in 1 mM Tris-HCl buffer (pH 8.0) for 1 hour (left), after which a fluorescence microscopy image was captured. After exposure to 250 μM of target anion (phosphate dibasic in this case) for 30 minutes, another image was captured (right). Comparison of the two images by visual inspection revealed a bead with moderate lighting-up response to the analyte (white arrow). Decoding of the sequence revealed the bead to be SHEY. See experimental methods for detailed description.

Table S2. MALDI-TOF mass spectrometry data for the resynthesized ODF sequences.

Sequence	Mass Expected	Mass Found	Sequence	Mass Expected	Mass Found
HYES	1474.51	1474.49	STKH	1656.6	1657.26
HYKS	1525.55	1532.84	STKY	1570.38	1570.64
HYKY	1725.61	1730.10	STTE	1650.39	1649.85
HYSE	1474.51	1474.71	STTS	1400.31	1400.15
SEHS	1274.45	1272.93	SYHS	1224.43	1223.69
SEKS	1289.29	2580.80[a]	SYKY	1439.33	1438.52
SHEY	1474.51	1473.13	SYTS	1269.26	1271.84
SHKY	1525.55	1528.47	SYTT	1600.37	1603.63
SHST	1355.48	1356.59	SYTY	1469.32	1469.44
SHSY	1224.43	1225.22	TTKS	1701.43	1704.24
SHYY	1424.49	1425.80	TTSY	1600.37	1602.24
SKST	1370.32	1369.60	TYEY	1719.40	1720.85
SSSE	988.16	987.92	TYYY	1669.38	1670.98
STHS	1355.48	1356.22	YYHE	1674.57	1675.57

[a] Dimer was observed.

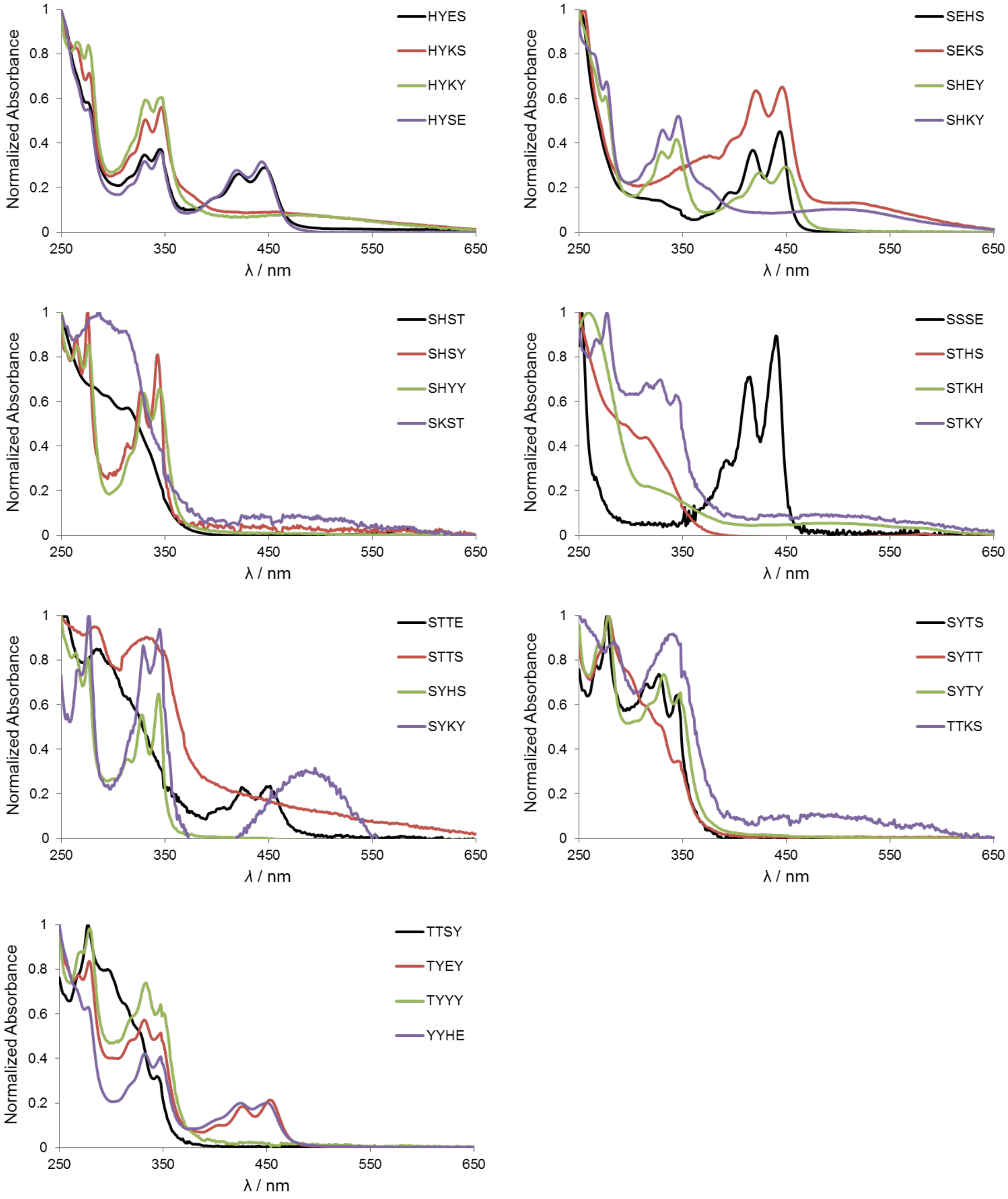

Figure S4. Absorption spectra of resynthesized ODF sequences (20 μM) in PBS buffer.

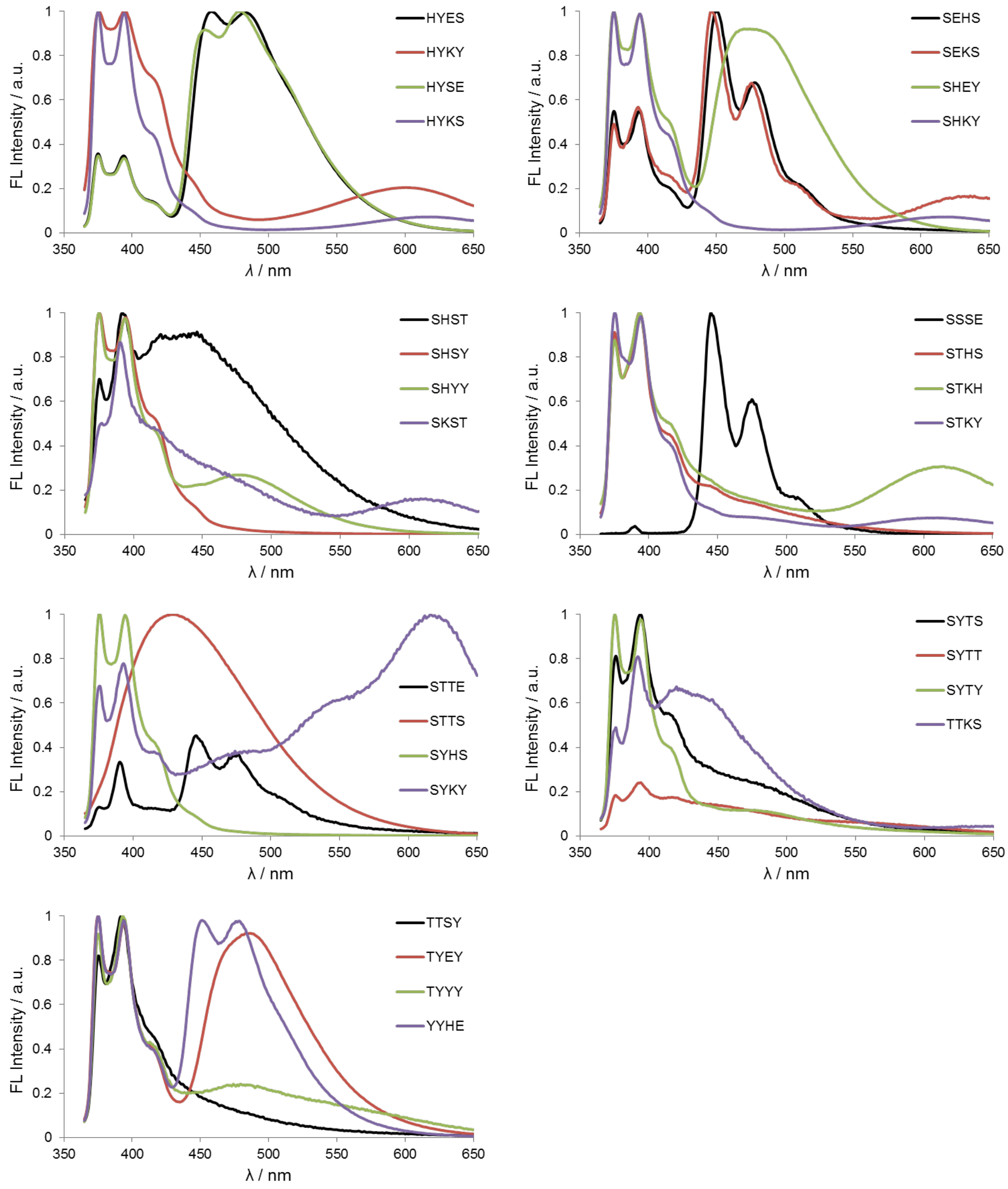

Figure S5. Fluorescence emission spectra of resynthesized ODF sequences (20 μM) in PBS buffer (λ_{ex} = 345 nm, λ_{em} > 365 nm).

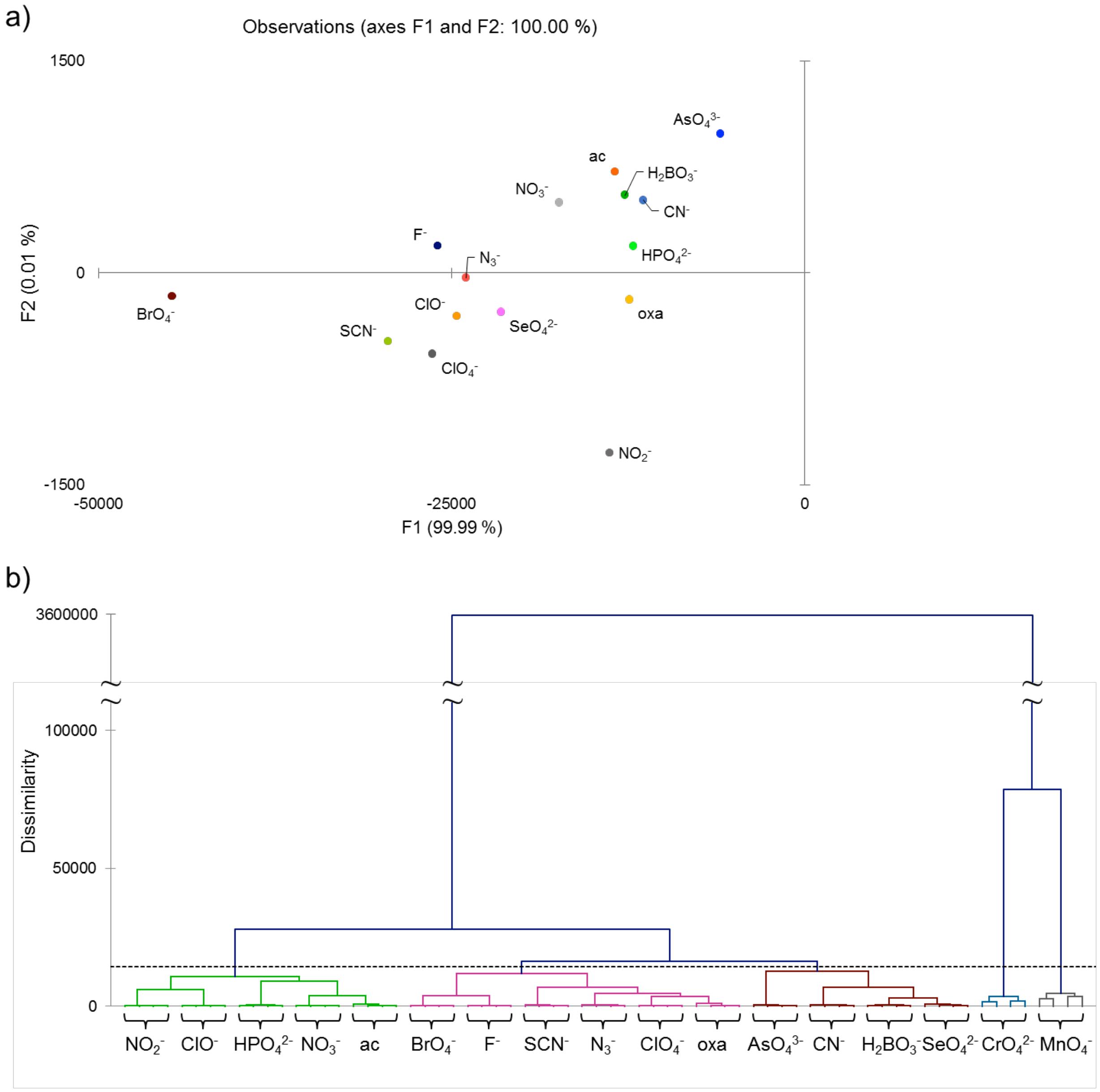

Figure S6. a) Discriminant analysis (DA) of twenty-nine screened ODF sensors versus seventeen anions at 250 μM in buffered water measuring ΔRGB. CrO_4^{2-} and MnO_4^- were well separated from the rest and not shown in the plot area. All seventeen anions were fully discriminated. Four individual replicate data points and 95% confidence ellipses were too small to be distinguished on this scale. b) Agglomerative hierarchical clustering (AHC) of the same data. All replicates were grouped together correctly.

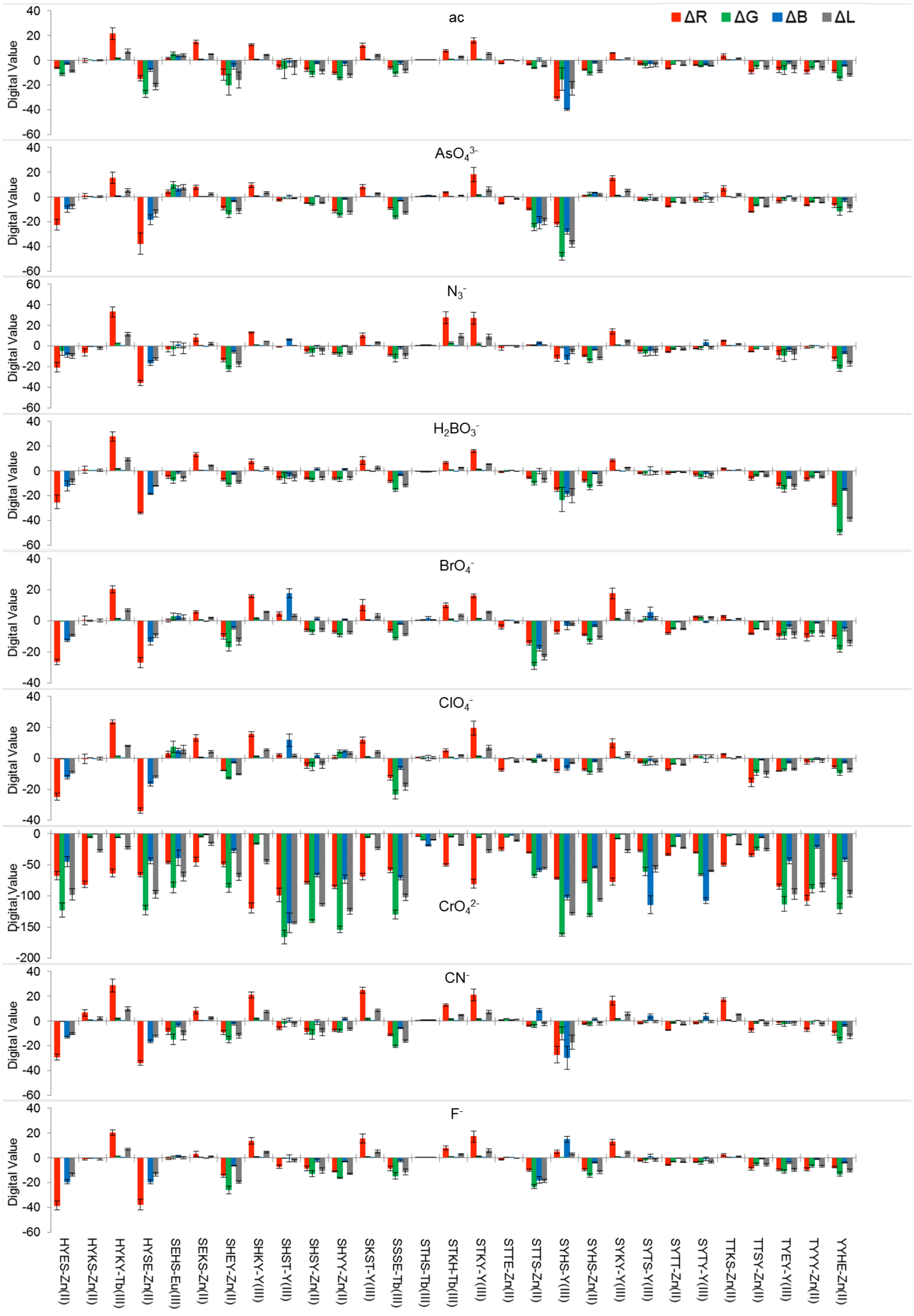
ac
ΔR ΔG ΔB ΔL
AsO_4^{3-}
N_3^-
$H_2BO_3^-$
BrO_4^-
ClO_4^-
CrO_4^{2-}
CN^-
F^-
Digital Value
HYES-Zn(II)
HYKS-Zn(II)
HYKY-Tb(III)
HYSE-Zn(II)
SEHS-Eu(III)
SEKS-Zn(II)
SHEY-Zn(II)
SHKY-Y(III)
SHST-Y(III)
SHSY-Zn(II)
SHYY-Zn(II)
SKST-Y(III)
SSSE-Tb(III)
STHS-Tb(III)
STKH-Tb(III)
STKY-Y(III)
STTE-Zn(II)
STTS-Zn(II)
SYHS-Y(III)
SYHS-Zn(II)
SYKY-Y(III)
SYTS-Y(III)
SYTT-Zn(II)
SYTY-Y(III)
TTKS-Zn(II)
TTSY-Zn(II)
TYEY-Y(III)
TYYY-Zn(II)
YYHE-Zn(II)

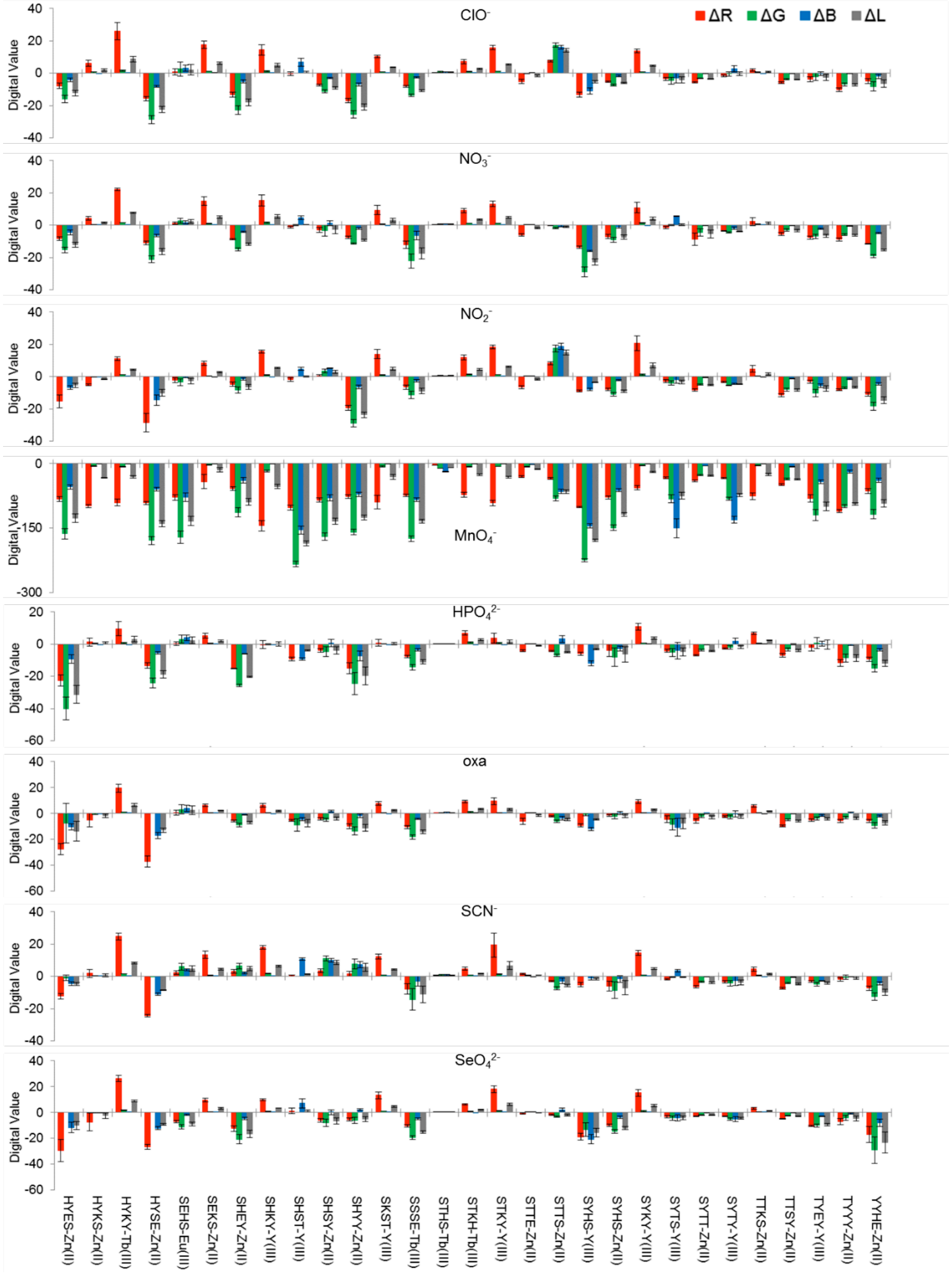

Figure S7. Quantitative fluorescence responses (as measured by ΔRGBL from microscopy images) of twenty-nine ODF sensors versus seventeen anions at 250 μM after 30 min equilibration in buffered water. Red, green, blue, and grey bars represent changes in R, G, B, and L (on a scale of ±255), respectively. The error bars represent standard deviation from four measurements.

a)

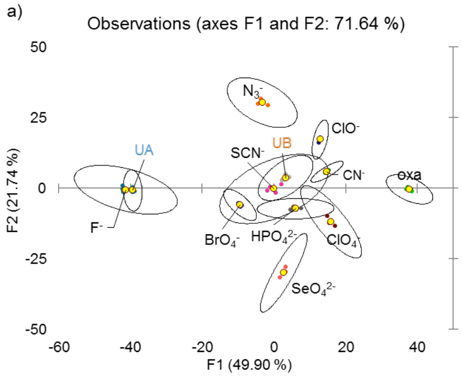

b)

from \ to	BrO_4^-	CN^-	ClO_4^-	F^-	HPO_4^{2-}	N_3^-	ClO^-	oxa	SCN^-	SeO_4^{2-}	UA	UB	Total	% correct
BrO_4^-	4	0	0	0	0	0	0	0	0	0	0	0	4	100.00%
CN^-	0	4	0	0	0	0	0	0	0	0	0	0	4	100.00%
ClO_4^-	0	0	4	0	0	0	0	0	0	0	0	0	4	100.00%
F^-	0	0	0	4	0	0	0	0	0	0	0	0	4	100.00%
HPO_4^{2-}	0	0	0	0	4	0	0	0	0	0	0	0	4	100.00%
N_3^-	0	0	0	0	0	4	0	0	0	0	0	0	4	100.00%
ClO^-	0	0	0	0	0	0	4	0	0	0	0	0	4	100.00%
oxa	0	0	0	0	0	0	0	4	0	0	0	0	4	100.00%
SCN^-	0	0	0	0	0	0	0	0	4	0	0	0	4	100.00%
SeO_4^{2-}	0	0	0	0	0	0	0	0	0	4	0	0	4	100.00%
UA	0	0	0	0	0	0	0	0	0	0	4	0	4	100.00%
UB	0	0	0	0	0	0	0	0	0	0	0	4	4	100.00%
Total	4	4	4	4	4	4	4	4	4	4	4	4	48	100.00%

Figure S8. Unknown anion identification test using the eight-sensor set. a) DA plot of ten anions plus two unknown samples. The anions tested were at 250 μM in buffered water, and the unknown samples (UA = F^-; UB = SCN^-) were measured in attempt to match with the standards. Ellipses represent 95% confidence levels around the centroids (yellow circles) of four replicate points. UA was confidently identified as fluoride based on this plot, but ambiguity arose for UB (although its centroid was closest to the centroid belonging to SCN^-). b) Confusion matrix analysis for the same data. See main text for explanation of the abbreviations.

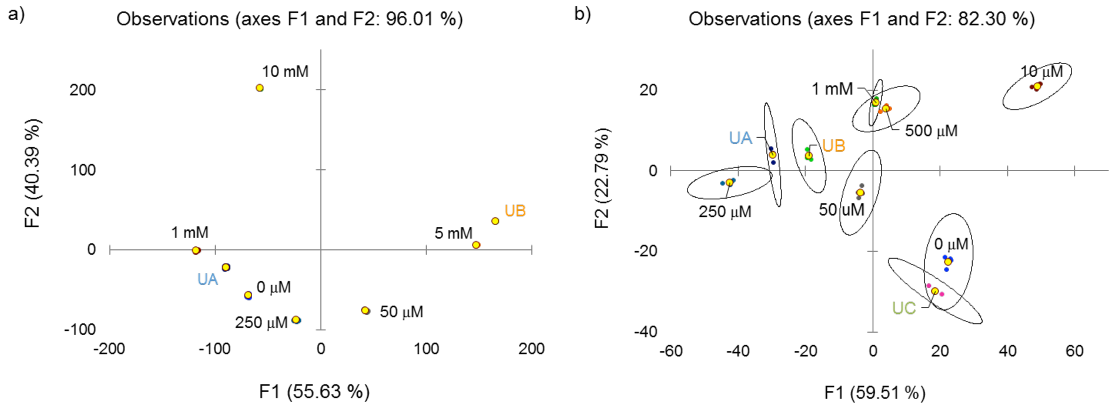

Figure S9. DA plots of samples of cyanide (left) and selenate (right) with unknown concentrations in buffered water. a) Based on proximity to centroids (yellow circles) belonging to standards (labeled by their concentration), UA was correctly identified as 1 mM and UB as 5 mM. 95% confidence ellipses were too small to be shown on this scale. b) Following the same procedure with smaller concentration range (0 to 1 mM), UA (250 μM) and UB (50 μM) were correctly identified using proximity to the standards. UC (10 μM) was incorrectly quantified as 0 μM. AHC analysis (Figure 5 in main text) showed similar results.

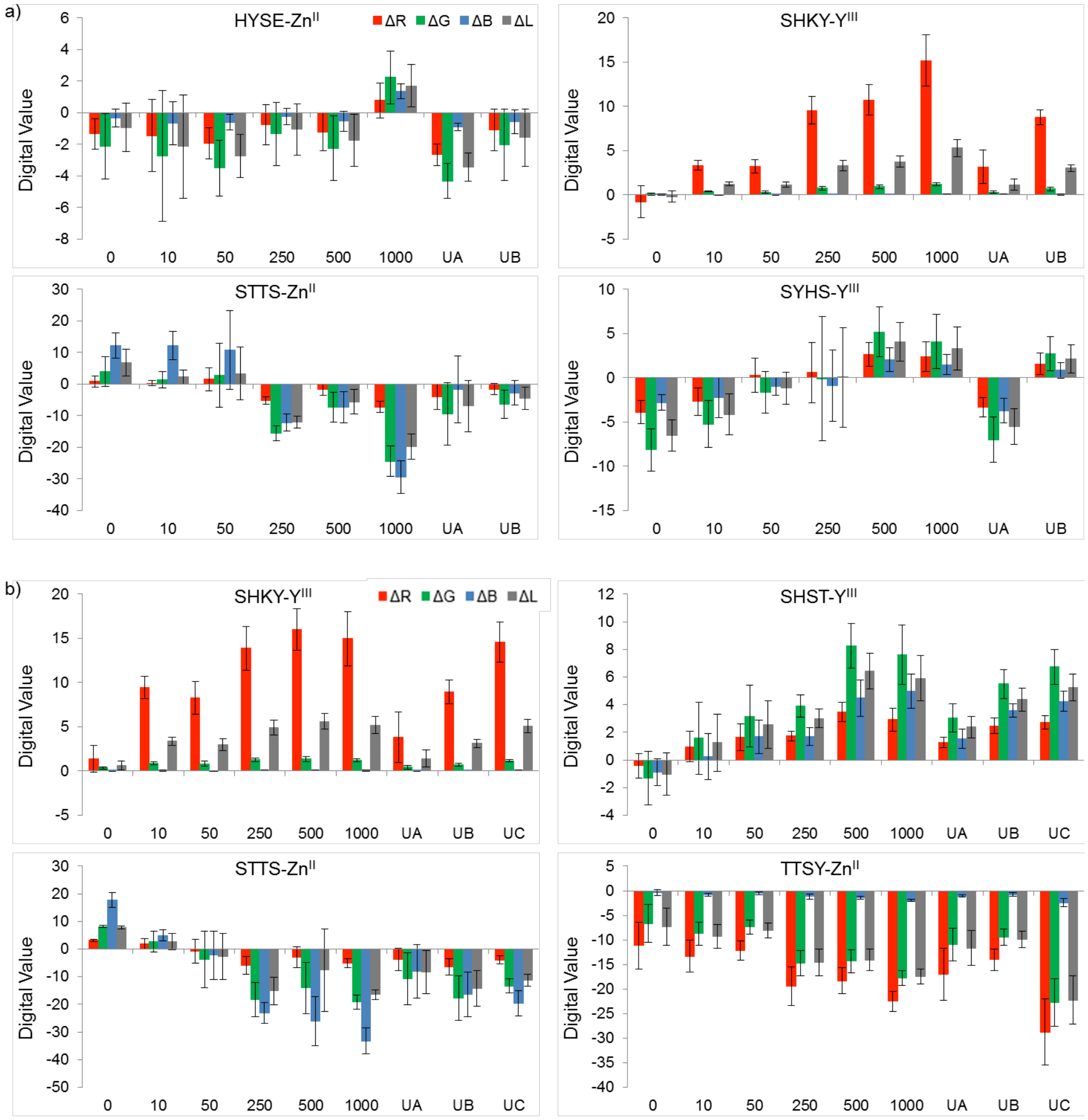

Figure S10. Quantitative fluorescence responses (ΔRGBL from microscopy images) from unknown concentration tests (value falling in between standards; from 0 to 1 mM) using four separate trials for a) SCN^- and b) AsO_4^{3-} in buffered water. Initially the eight-sensor set was used to measure the response but only four was used in subsequent statistical analysis (see figures for list) as they produced relatively linear response as function of concentration. Data for standards were labelled by the value of their concentration in micromolar. Red, green, blue, and grey bars represent changes in R, G, B, and L (on a scale of ±255), respectively, and the error bars represent standard deviation. For a), UA = 300 μM and UB = 30 μM. For b), UA = 5 μM, UB = 20 μM, and UC = 800 μM.

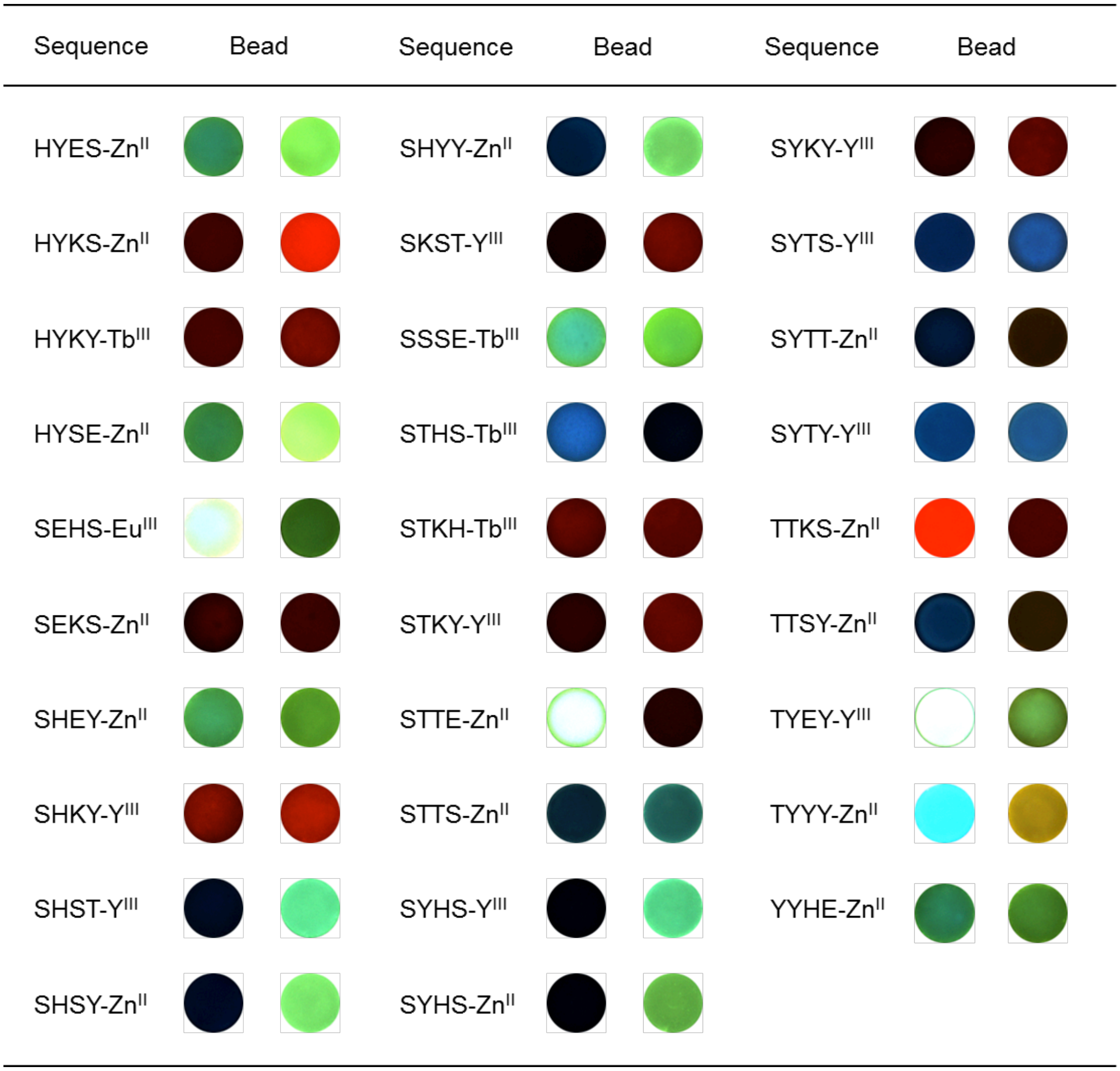

Figure S11. Sample images of twenty-nine resynthesized ODF tetramer sequences on PS microbeads using an epifluorescence microscope (λ_{ex} = 340-380 nm, λ_{em} > 420 nm). The beads on the left side of each column are beads containing ODFs without added metal, and those on the right are after exposure to one of four (Eu^{III}, Tb^{III}, Y^{III}, or Zn^{II}) metal nitrate salts (see Experimental Methods for details). All beads were imaged after incubation in 1 mM Tris-HCl buffer (pH 8) for one hour at room temperature. Each bead represents the average color of the batch. See Figure 1A in main text for structures of the ODF monomers (listed in 5'→3' direction).

References

[1] J. Gao, C. Strässler, D. Tahmassebi, E. T. Kool, *J. Am. Chem. Soc*. **2002**, *124*, 11590-11591

[2] M. H. Ohlmeyer, M. Swanson, L. W. Dillard, J. C. Reader, G. Asouline, R. Kobayashi, M. Wigler, W. C. Still, *Proc. Natl. Acad. Sci. U. S. A*. **1993**, *90*, 10922-10926; H. P. Nestler, P. A. Barlett, W. C. Still, *J. Org. Chem*. **1994**, *59*, 4723-4724.

[3] S. Wang, J. Guo, T. Ono, E. T. Kool, *Angew. Chem. Int. Ed*. **2012**, *51*, 7176-7180.
